# Radiopharmacokinetic modelling and radiation dose assessment of ^223^Ra used for treatment of metastatic castration-resistant prostate cancer

**DOI:** 10.1186/s40658-021-00388-1

**Published:** 2021-06-02

**Authors:** Vera Höllriegl, Nina Petoussi-Henss, Kerstin Hürkamp, Juan Camilo Ocampo Ramos, Wei Bo Li

**Affiliations:** 1 Institute of Radiation Medicine, Helmholtz Zentrum München, German Research Center for Environmental Health, Ingolstädter Landstraße 1, 85764 Neuherberg, Germany; 2 Department of Medical Physics, Memorial Sloan Kettering Cancer Center, 1250 First Avenue, New York, NY 10065 USA

**Keywords:** Radiopharmaceutical, Biokinetic models, ^223^Ra, Internal dose, Radionuclide therapy

## Abstract

**Purpose:**

Ra-223 dichloride (^223^Ra, Xofigo®) is used for treatment of patients suffering from castration-resistant metastatic prostate cancer. The objective of this work was to apply the most recent biokinetic model for radium and its progeny to show their radiopharmacokinetic behaviour. Organ absorbed doses after intravenous injection of ^223^Ra were estimated and compared to clinical data and data of an earlier modelling study.

**Methods:**

The most recent systemic biokinetic model of ^223^Ra and its progeny, developed by the International Commission on Radiological Protection (ICRP), as well as the ICRP human alimentary tract model were applied for the radiopharmacokinetic modelling of Xofigo® biodistribution in patients after bolus administration. Independent kinetics were assumed for the progeny of ^223^Ra. The time activity curves for ^223^Ra were modelled and the time integrated activity coefficients, $\overset{\sim }{a}\left({r}_S,{T}_D\right),$ in the source regions for each progeny were determined. For estimating the organ absorbed doses, the Specific Absorbed Fractions (SAF) and dosimetric framework of ICRP were used together with the aforementioned $\overset{\sim }{a}\left({r}_S,{T}_D\right)$ values.

**Results:**

The distribution of ^223^Ra after injection showed a rapid plasma clearance and a low urinary excretion. Main elimination was via faeces. Bone retention was found to be about 30% at 4 h post-injection. Similar tendencies were observed in clinical trials of other authors. The highest absorbed dose coefficients were found for bone endosteum, liver and red marrow, followed by kidneys and colon.

**Conclusion:**

The biokinetic modelling of ^223^Ra and its progeny may help to predict their distributions in patients after administration of Xofigo®. The organ dose coefficients of this work showed some variation to the values reported from clinical studies and an earlier compartmental modelling study. The dose to the bone endosteum was found to be lower by a factor of ca. 3 than previously estimated.

**Supplementary Information:**

The online version contains supplementary material available at 10.1186/s40658-021-00388-1.

## Introduction

Worldwide, prostate cancer is the second most frequent cancer in men [1]. In 2013, ^223^Ra-dichloride (^223^Ra, Xofigo®, Bayer) was approved by the US Food and Drug Administration (FDA) and by the European Medicines Agency (EMA) as a tolerated radiopharmaceutical for treatment of patients suffering from castration-resistant prostate cancer with bone metastases and no visceral metastases [2]. The bone metastases can result in severe bone pain and symptoms like pathologic fractures, spinal cord compression or myelosuppression [3]. Radium (Ra, ^223^Ra) behaves similar to calcium after its intravenous injection into the human body and its main target is the bone at areas of active bone formation, thereby forming complexes with the bone mineral hydroxyapatite [4]. ^223^Ra is an alpha-emitter (physical half-life 11.4 days) with a high linear energy transfer (80 keV/μm) [2] which may lead to a high frequency of double-strand DNA breaks in tumour cells; this may result in a highly localised cytotoxic effect to tumour cell death. The short alpha particle path range (< 100 μm) [2] may minimise damage to the surrounding normal tissue. After intravenous administration of ^223^Ra, its bone-seeking and alpha-particle emitting properties may reduce bone pain, thereby improving the quality of life of the patients. The phase III ALSYMPCA (ALpharadin in SYMPtomatic Prostate CAncer) study [5] demonstrated that the treatment with ^223^Ra extends the overall survival time of patients versus placebo by 3.6 months. ^223^Ra decays via six short-lived daughter nuclides into the stable lead isotope ^207^Pb (Table 1) [6]. The total emitted energy is 28.2 MeV, of which 95.3% is from alpha emission, 3.6% from beta and 1.1% from gamma emission. Although the photon yield is low, gamma camera imaging is feasible [7]. In the last years, several clinical trials using Xofigo® were performed in order to demonstrate the pharmacokinetic behaviour of ^223^Ra in the human body after its intravenous injection and to estimate organ absorbed doses in radiation-sensitive organs and tissues, such as bone surface and red marrow [8–11]. In 2017, the International Commission on Radiological Protection (ICRP) published new biokinetic data of ^223^Ra and its progeny in ICRP Publication 137 [12]. The improved systemic model of radium is a modification of the previous model of ICRP Publication 67 [13]. Revisions have been made to provide more physiologically based data of uptake and retention of radium and other radionuclides in organs and tissues. After intake of radium either by inhalation or ingestion, radium gradually enters the blood and is transferred to all parts of the body [14, 15]. It can be assumed that the ICRP systemic model for workers and members of the public applies not only to ingestion but (also) to materials taken up by blood in general. For example, the model is applicable to the ingestion of pure radium due to oral intake of ^226^Ra or ^224^Ra (in the sulphate form) from mock radium dial paint [14, 15], as well as to the intravenous injection of ^223^RaCl_2_ as radiopharmaceutical. Although ^224^Ra-sulphate is an insoluble inorganic salt, studies of the so-called “watch dial painters” showed that it could be absorbed from the alimentary tract by a factor of 0.2 [16]. The extent of uptake of radium into blood is dependent on the chemical compound, but not the behaviour of radium once taken up to the blood. Radium entering the bloodstream by either direct injection or ingestion shows similar biokinetic behaviour in the systemic circulation and is transferred to all parts of the body and especially in the bones. Therefore, it can be assumed that the ICRP biokinetic models are valid not only for application in radiation protection of the workers but also to predict the distribution of radium and its progeny for a reference patient in nuclear medicine.

**Table 1:** Decay chain of ^223^Ra and its progeny to stable ^207^Pb (from [6])

Radionuclide	Decay mode	Abundance (%)	Half-life
^223^Ra ➔ ^219^Rn	α	100	11.43 d
^219^Rn ➔ ^215^Po	α	100	3.96 s
^215^Po ➔ ^211^Pb	α	100	1.78 ms
^211^Pb ➔ ^211^Bi	β^-^	100	36.1 min
^211^Bi ➔ ^211^Po	β^-^	0.276	2.14 min
^211^Bi ➔ ^207^TI	α	99.72	2.14 min
^211^Po ➔ ^207^Pb	α	100	0.516 s
^207^TI ➔ ^207^Pb	β^-^	100	4.77 min

The objective of this work is to apply the new biokinetic model of ICRP for radium and its progeny, follow their distribution in the human body and compare the model prediction with data of patients reported during four clinical trials [8, 10, 11, 17]. Moreover, the organ absorbed dose coefficients after intravenous injection of ^223^Ra were estimated according to the ICRP/MIRD schema for radiopharmaceutical dosimetry [18] by applying the latest SAF values of ICRP; these were calculated for the male reference adult following the ICRP current methodology [19]. The results were then compared with organ absorbed doses estimated via imaging data of patients, and additionally with a compartmental modelling study of Lassmann and Nosske [20] who used the biokinetic model of ICRP Publication 67 [13] and earlier dosimetric methods.

## Materials and methods

### Radiopharmacokinetic modelling

**Fig. 1:**
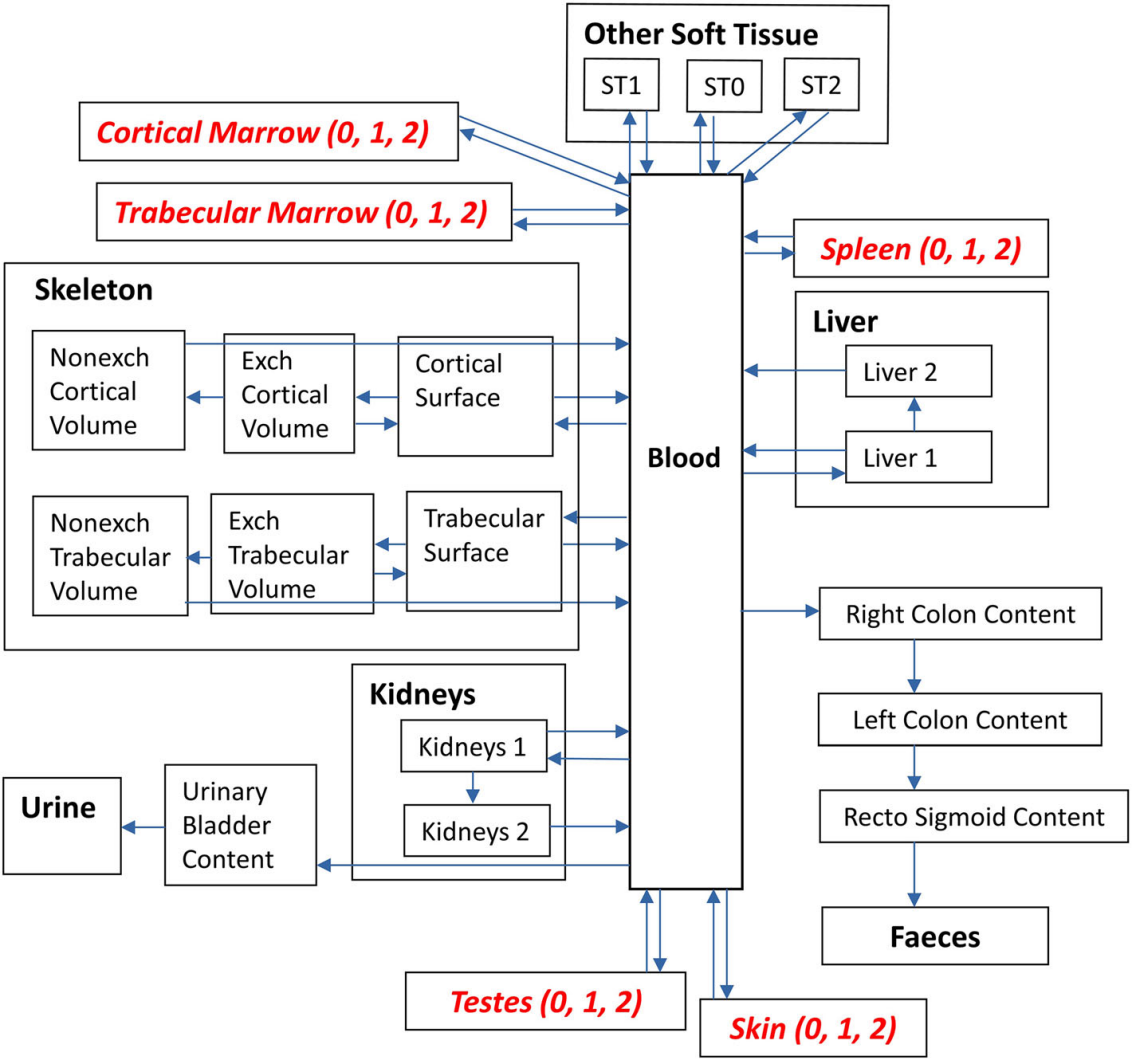
Systemic model of radium ^223^Ra used in the present work for the biokinetic and dosimetric modelling. It is based on the respective model given in ICRP Publication 137 [12] and has been modified to include extra compartments for *Cortical Marrow*, *Trabecular Marrow*, *Spleen*, *Skin* and *Testes*, indicated in italic. Exch, exchangeable; nonexch, non-exchangeable; ST, soft tissue. The indication (0, 1, 2) refers to fast, intermediate and slow turnover

ICRP Publication 137 [12] provides a modification of the radium model of ICRP Publication 67 [13] with new improved biokinetic data of ^223^Ra and its progeny. Figure 1 shows the modified Ra (^223^Ra) model adopted in this work for pharmacokinetic modelling and dosimetry and Table 2 lists the transfer coefficients used for this model. The systemic models of the ^223^Ra progeny and the respective transfer coefficients (d^−1^) are shown in detail in the Supplementary Material.

**Table 2:** Model parameters of ^223^Ra: Transfer coefficients *k* (per day) taken from ICRP *Publications* 100 [21] and 137 [12]. Please note that the transfer rates from the compartment *Blood* to *Other Soft Tissue* (*ST0*, *ST1*, *ST2*) are lower than the values given in [12] because of the added compartments *Trabecular Marrow*, *Cortical Marrow*, *Spleen*, *Skin* and *Testes*

From	To	*k* (d^−1^)
Blood	ST0	18.412
Blood	ST1	3.079
Blood	ST2	0.062
Blood	Cortical bone surface	7.78
Blood	Trabecular bone surface	9.72
Blood	Kidneys 1	1.4
Blood	Liver 1	4.2
Blood	Right colon content	21.79
Blood	Urinary bladder content	0.606
Liver 1	Blood	0.691
Liver 2	Blood	0.0019
Liver 1	Liver 2	0.00208
ST0	Blood	6.98
ST1	Blood	0.693
ST2	Blood	0.00038
Cortical bone surface	Blood	0.578
Cortical bone surface	Exch cortical bone volume	0.116
Exch cortical bone volume	Cortical bone surface	0.0185
Exch cortical bone volume	Nonexch cortical bone volume	0.0046
Nonexch cortical bone volume	Blood	0.0000821
Trabecular bone surface	Blood	0.578
Trabecular bone surface	Exch trabecular bone volume	0.116
Exch trabecular bone volume	trabecular bone surface	0.0185
Exch trabecular bone volume	Nonexch trabecular bone volume	0.0046
Nonexch trabecular bone volume	Blood	0.000493
Trabecular marrow 0	Blood	6.98
Trabecular marrow 1	Blood	0.693
Trabecular marrow 2	Blood	0.00038
Blood	Trabecular marrow 0	1.190
Blood	Trabecular marrow 1	0.199
Blood	Trabecular marrow 2	0.00398
Cortical marrow 0	Blood	6.98
Cortical marrow 1	Blood	0.693
Cortical marrow 2	Blood	0.00038
Blood	Cortical marrow 0	0.098
Blood	Cortical marrow 1	0.016
Blood	Cortical marrow 2	0.00033
Blood	Skin 0	1.165
Blood	Skin 1	0.195
Blood	Skin 2	0.0039
Blood	Spleen 0	0.053
Blood	Spleen 1	0.0089
Blood	Spleen 2	0.00018
Blood	Testes 0	0.0124
Blood	Testes 1	0.0021
Blood	Testes 2	0.000041
Skin 0	Blood	6.98
Skin 1	Blood	0.693
Skin 2	Blood	0.00038
Spleen 0	Blood	6.98
Spleen 1	Blood	0.693
Spleen 2	Blood	0.00038
Testes 0	Blood	6.98
Testes 1	Blood	0.693
Testes 2	Blood	0.00038
Kidneys 1	Blood	2.073
Kidneys 1	Kidneys 2	0.00624
Kidneys 2	Blood	0.0019
Right colon content	Left colon content	2.0
Left colon content	Recto sigmoid content	2.0
Recto sigmoid content	Faeces	2.0
Urinary bladder content	Urine	12.0

*Exch*, exchangeable; *nonexch*, non-exchangeable; *ST*, soft tissue

The indication (0, 1, 2) refers to fast, intermediate and slow turnover

The systemic model of ^223^Ra consists of main compartments representing the *Skeleton*, *Blood*, *Liver* and *Kidneys* with excretion pathway to *Urine* and the large intestine parts of the *Alimentary Tract content* with excretion pathway to *Faeces*. A compartment of *Other Soft Tissue* is linked to the transfer compartment *Blood. Other Soft Tissue* comprises all soft tissues, which were not explicitly defined in the systemic model of Ra, with three sub-compartments ST0, ST1 and ST2 for fast, intermediate and slow turnover, respectively. The compartments *Skeleton*, *Liver and Kidneys* and the *Alimentary Tract content* are also subdivided. The earlier version of the ^223^Ra model [13] featured only one liver compartment and no compartment for kidneys, as the latter was part of the *Other Soft Tissue*. In order to incorporate more physiologically based data of the biokinetics of Ra, a kidney compartment was inserted and both *Kidneys* and *Liver* are modelled as two compartments representing slow and fast turnover of radium. The *Skeleton* has separate sub-compartments for cortical and trabecular bone, and each is compartmentalised into bone surface and bone volume; the latter is again divided into a non-exchangeable (nonexch) and exchangeable (exch) part. The large intestine includes, according to the Human Alimentary Tract Model (HATM) [21], the contents of the *Right Colon* (*RC*), *Left Colon* (*LC*) and * Recto Sigmoid* (*RS*). It should be noted that a transfer from the compartment for *Small Intestine* (*SI*) *content* to the *Blood c*ompartment is not included in the systemic Ra model because there is no transfer flow from the systemic compartments into SI or upper compartments of the alimentary tract.

Each compartment is linked through the decay constant of ^223^Ra with the corresponding compartments of the next progeny, ^219^Rn. As the biokinetics of the ^223^Ra progeny are independent of those of the parent nuclide, their transfer rates and compartment structures are not necessarily identical to those of their parent. Compartments representing the organs/tissues *Spleen*, *Skin*, *Testes*, *Trabecular Marrow* and *Cortical Marrow* are specific for some biokinetic models of the daughter nuclides. Therefore, prior to the complete biokinetic modelling of ^223^Ra and its progeny and according to ICRP 137 [12], the biokinetic model of each nuclide of the decay chain (including ^223^Ra) was expanded to the five complementary compartments mentioned above; all transfer rates were taken from ICRP 137 [12]. The transfer rates from the *Blood* compartment to the newly established compartments of *Trabecular Marrow*, *Cortical Marrow*, *Spleen*, *Skin* and *Testes* were calculated from the corresponding transfer rate of the *Other Soft Tissue* compartment by considering their mass-fraction. As the latter five compartments are part of the *Other Soft Tissue* compartment of the original ICRP 137 [12] Ra model, their kinetics should be identical to the *Other Soft Tissue* kinetics. Therefore, three additional compartments for each of the five newly established compartments were integrated into the Ra model, with fast (indicated by a ‘0’), intermediate (‘1’) and slow (‘2’) turnover (see Fig. 1). As part of a decay series, each of the three sub-compartments was connected to the corresponding single compartment of radon by its decay constant. The masses of the abovementioned organs and tissues were taken from ICRP Publication 133 [19]. The transfer rate from the *Blood* compartment to the *Other Soft Tissue* was reduced accordingly.

### Implementation of the compartmental model in SAAM II software

The biokinetics of ^223^Ra and its progeny were described by a system of first-order linear ordinary differential equations, which were numerically solved by using the commercial software package SAAM II, version 2.3^1^ [22]. Model parameters (compartmental structures and transfer rates) of ^223^Ra and its daughter nuclides (^219^Rn, ^215^Po, ^211^Pb, ^211^Bi, ^211^Po and ^207^TI) were used as input. The equivalent compartments of each model element were connected through their decay constants, which were calculated from the half-lives (see Table 1). In order to follow the time-dependent distribution of ^223^Ra and its progeny in the organs and tissues as well as the excretion path, a period of 168 h (7 days) after simulated injection was assumed. This time period was selected for comparison purposes with the clinical studies of Yoshida et al. [8], Chittenden et al. [10] and Carrasquillo et al. [11] comprising data measured for this particular period. For dose assessment, the time-integrated activity coefficients were calculated as the area under the curve (AUC) up to a time period of 1000 days after injection, since beyond this time the AUC in each organ and tissue remain unchanged.

### Calculation of absorbed dose coefficients

The determination of internal doses can be performed by mathematical calculations using standardised biokinetic and dosimetric models. The ICRP and the Committee on Medical Internal Radiation Dose (MIRD) of Society of Nuclear Medicine generalised the schema for the calculation of absorbed dose to patients from administered radiopharmaceuticals [18]. The absorbed dose coefficient is calculated by the time integrated activity coefficients $\overset{\sim }{a}\left({r}_S,{T}_D\right)$ of a radionuclide in source organs, and S values describing the mean absorbed dose rate to target tissue *r*_*T*_ per unit activity present in source tissue *r*_*S*_. The S values are based on the Specific Absorbed Fractions (SAF), defined as the fraction of energy *E*_*R*, *i*_ of radiation type *R* emitted within the source tissue *r*_*S*_ that is absorbed per mass in the target tissue *r*_*T*_ (kg^−1^).

For this work, the SAFs were taken from the ICRP Publication 133 [19] and have been calculated for the ICRP adult male reference voxel phantom [23] using Monte Carlo methods. For some small tissues beyond the resolution of the voxel phantom, as for the cortical and trabecular bone surface, the alimentary and respiratory tracts, other detailed models were used.

## Results

Time integrated activity coefficients, $\overset{\sim }{a}\left({r}_S,{T}_D\right),$ for each radionuclide and each source region were calculated and are listed in Table 3. An in-house dose computational software [24, 25] was used which incorporates the activity coefficients, the SAF values and the most recent nuclear decay data of ICRP Publication 107 [6] and performs dose calculations for photons, electrons and alpha particles of the desired radionuclide. The absorbed dose to a target region was estimated by summing the contributions from the decay of the daughter products of ^223^Ra i.e. ^219^Rn, ^215^Po, ^211^Pb, ^211^Bi, ^211^Po and ^207^TI.

**Table 3:** Time integrated activity coefficients, $\overset{\sim }{a}\left({r}_S,{T}_D\right),$ for each radionuclide and each source region (in h)

Source region	^223^Ra	^219^Rn	^215^Po	^211^Pb	^211^Bi	^211^Po	^207^TI
Blood	0.89	1.00	1.00	1.11	1.02	0.003	1.01
Other tissues	6.85	6.84	6.84	6.71	6.70	0.02	6.88
Cortical bone surface	9.49	9.42	9.42	9.18	9.18	0.03	9.08
Cortical bone volume	14.14	14.14	14.14	14.30	14.30	0.04	14.26
Trabecular bone surface	11.85	11.77	11.77	11.47	11.47	0.03	11.33
Trabecular bone volume	17.66	17.66	17.66	17.86	17.86	0.05	17.81
Cortical marrow	0.04	0.04	0.04	0.04	0.04	0.0001	0.04
Trabecular marrow	0.44	0.44	0.44	0.45	0.45	0.001	0.45
Kidneys	0.64	0.64	0.64	0.68	0.72	0.002	0.73
Urinary bladder content	0.04	0.04	0.04	0.06	0.08	0.0002	0.08
Liver	5.12	5.11	5.11	5.19	5.22	0.01	5.17
Skin	0.43	0.43	0.43	0.44	0.44	0.001	0.44
Spleen	0.02	0.02	0.02	0.02	0.02	0.0001	0.02
Testes	0.005	0.005	0.005	0.005	0.005	0.0000	0.006
Small intestine content			0.000	0.005	0.007	0.0000	0.002
Right colon content	9.40	9.40	9.40	8.78	8.74	0.02	8.64
Left colon content	9.12	9.12	9.12	9.10	9.10	0.03	9.07
Recto sigmoid content	8.85	8.85	8.85	8.87	8.87	0.03	8.85

**Fig. 2:**
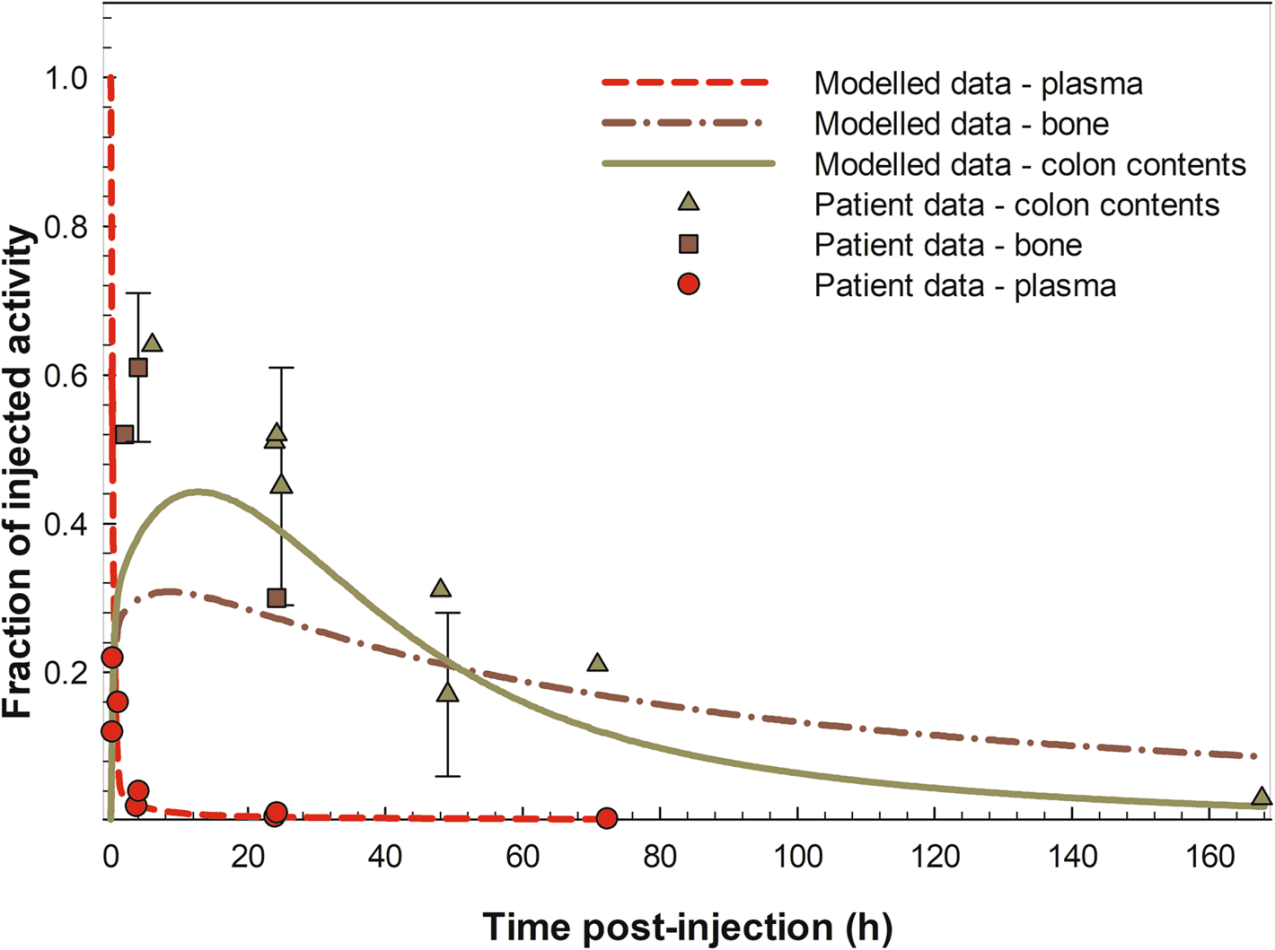
Time-dependent distribution of ^223^Ra in plasma, bone and colon after intravenous injection, as modelled for the present work. Patient data from [8, 10, 11, 17] are also shown with median values or values of mean ± SD. Bone indicates total bone, i.e. cortical and trabecular surface, volume and bone marrow

**Fig. 3:**
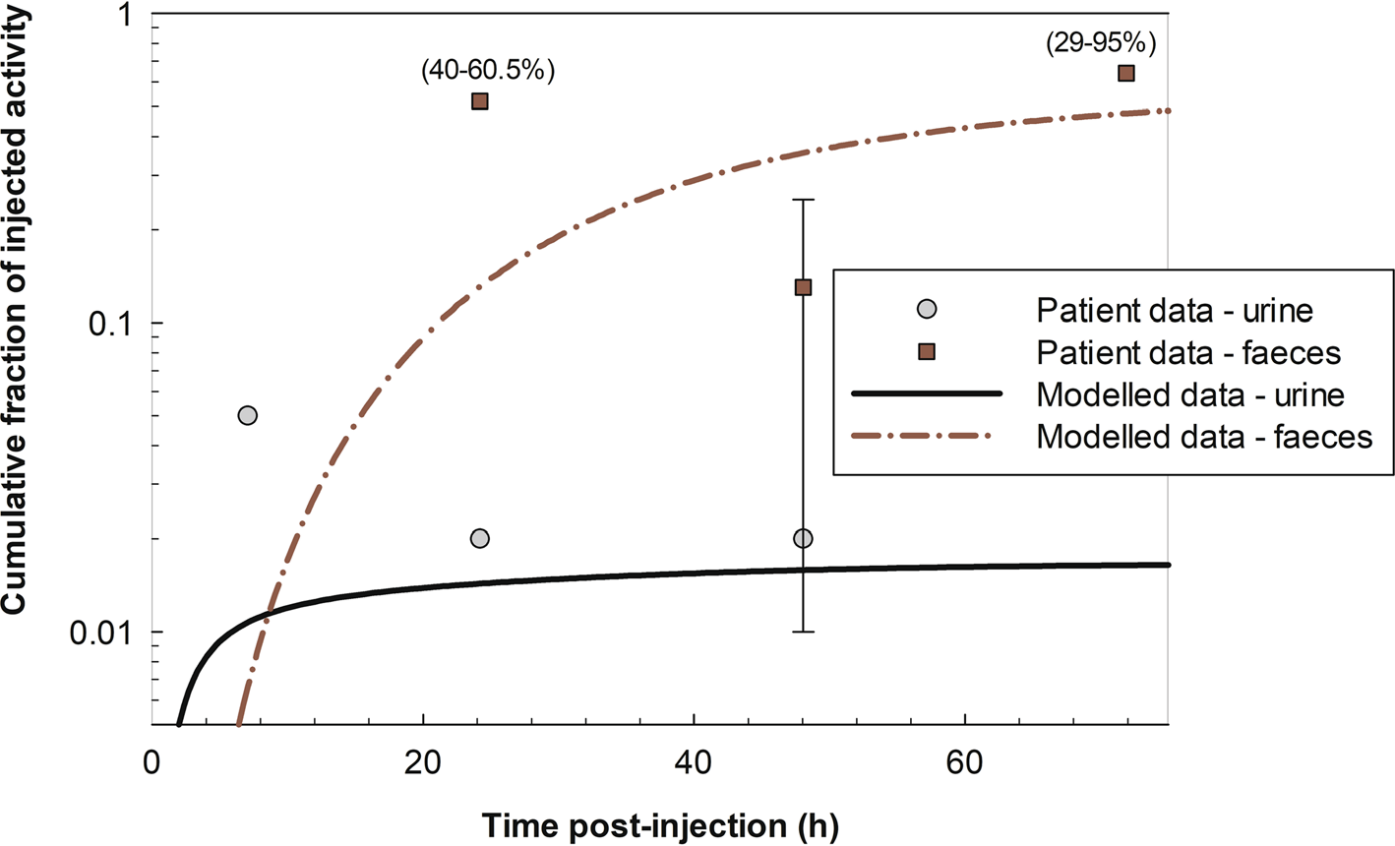
Cumulative urinary and faecal excretion of ^223^Ra after intravenous injection as modelled for the present work. Data of patients from three clinical trials [8, 10, 11] are shown with mean values or mean ± SD, or ranges (in parentheses)

Figure 2 shows the time-dependent distribution of ^223^Ra after a simulated intravenous administration, as modelled for this work. Data from four clinical trials [8, 10, 11, 17] with in total 47 patients receiving Xofigo® due to bone metastases are also shown. It can be seen that the plasma clearance of ^223^Ra was very fast: 2 h after injection about 2.5% of the injected activity remained in the plasma and after 24 h post-injection, less than 1%. Initial bone uptake of ^223^Ra within 4 h post-injection was about 30%, primarily into the bone surface (both cortical and trabecular). A slow uptake of up to 6% of ^223^Ra was found into the bone volume (both cortical and trabecular) at 80 h post-injection (data not shown). Figure 3 shows the cumulative urinary and faecal excretion of ^223^Ra after intravenous injection as modelled for the present work. Patient data from three clinical trials [8, 10, 11] are also shown. Whilst the cumulative urinary excretion was low (ca. 2%), the cumulative excretion into faeces amounted to about 50% after 3 days post-injection; therefore, the main elimination of ^223^Ra was through colon (into faeces) and not through the urinary pathway.

**Fig. 4:**
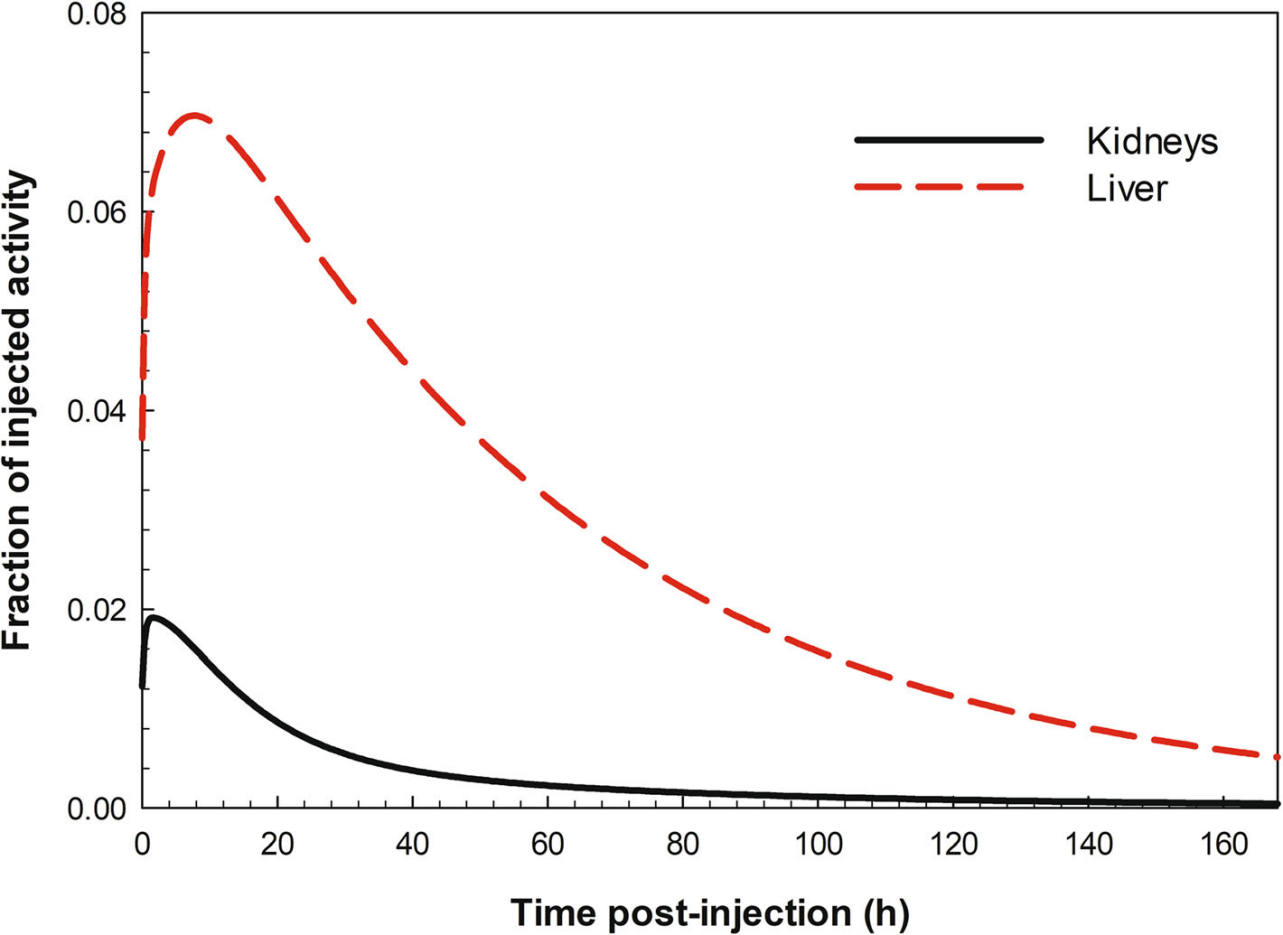
Modelled time-dependent distribution of ^223^Ra in kidneys and liver after intravenous injection

Figure 4 reveals a low uptake in the liver, up to 7% after 7 h post-injection, and a ca. 2% uptake in kidneys at 2 h after injection. No significant distribution of ^223^Ra was observed in other organs or tissues, neither for radium progeny. Table 4 summarises the modelled data, together with the clinical data showing the wide range of distribution patterns of ^223^Ra in the body of the patients. It should be noted that the subdivision of the colon as part of the human alimentary tract is differently defined for the HATM [21] as for the GI tract [26] model.

**Table 4:** Overview of the biodistribution of ^223^Ra after intravenous injection in patients from four clinical trials [8, 10, 11, 17] in comparison to the modelled data of the present work; data expressed as percentage of administered ^223^Ra; values given as range, median or mean ± SD; *n* = patient number

Region	Time post-injection	Present work	Nilsson et al. [17] *n* = 25	Carrasquillo et al. [11] *n* = 10	Chittenden et al. [10] *n* = 6	Yoshida et al. [8] *n* = 6
Plasma	10-15 min	55	12			9-28 (22)
	4 h	2.0		1.6-3.9 (2)		1-6 (4)
	24 h	0.5	< 1	0.37-1.0 (0.55)	0.6-5.1 (1.1)	
	3 d	0.2				0-0.9 (0.3)
Bone^a^	2 h	28.2				41-57 (52)
	4 h	29.8			61 ± 10	
Urine (cumulative)	4 h	0.83		5 (first void)		
	2 d	1.58			2 ± 2	2
Faeces (cumulative)	1 d	12.9		40-61 (52)		
	2 d	35.3			13 ± 12	
	3 d	47.4				29-95 (64)
	7 d	52.1			60-80	
RC/ULI* content	1 d	11.6			45 ± 16	
	7 d	0.53			0-18 (4)	
LC/LLI** content	2 d	7.4			17 ± 11	
	7 d	0.64			6 ± 4	
HATM/GI tract*** content	6 h	40.9				22-85 (64)
	1 d	39.3		50.8 ± 7.5		32-78 (52)
	2 d	22.0				4-76 (31)
	3 d	11.8				5-53 (21)
	7-8 d	1.9				0-9 (3)

^a^Total bone, i.e. surface, volume and bone marrow

*RC/ULI, right colon/upper large intestine

**LC/LLI, left colon/lower large intestine

***HATM, human alimentary tract model (HATM) [21] referring in the present model to the RC, LC and recto sigmoid; *GI tract*, gastrointestinal tract (ICRP 30, [26]) representing small intestine, ULI and LLI. The present work refers to RC/LC/HATM content, whereas the clinical studies refer to ULI/LLI/GI tract content

### Absorbed dose coefficients

From the biokinetic modelling, 17 source regions were defined: blood, cortical and trabecular bone surfaces, cortical and trabecular bone volumes, cortical and trabecular marrow, kidneys, urinary bladder content, liver, testes, spleen, skin, right colon content, left colon content, recto sigmoid content and other tissues. Time integrated activity coefficients for each radionuclide and each source region as calculated by the biokinetic model can be found in Table 3. Absorbed doses for 28 target organs were calculated. Table 5 presents organ absorbed dose coefficients (in mGy/MBq) for some selected organs, considering the alpha, beta, and gamma contribution to the total dose. The dosimetric method used is the one described in [19]. The highest doses were observed in the endosteum (endosteal cells), red marrow, liver, kidneys and colon. Table 5 also shows the values of a previous modelling study of Lassmann and Nosske [20] and of the clinical studies of Chittenden et al. [10] and Yoshida et al. [8]. The results of the present work for red marrow and endosteum showed lower dose values (by a factor 2.3-3.4), compared to the modelling study of Lassmann and Nosske [20] and the clinical study of Yoshida et al. [8] and even lower by a factor of 13-25 to values of the clinical study of Chittenden et al. [10]. However, the dose coefficients for liver and kidneys were found to be much higher than those of the clinical studies.

**Table 5:** Absorbed dose coefficients (mGy/MBq) with contribution from alpha, beta and gamma radiation to the total absorbed dose, after simulated injection of ^223^Ra, in comparison to the modelling study of Lassmann and Nosske [20] and the clinical studies of Chittenden et al. [10] and Yoshida et al. [8]. Values of Yoshida et al. refer to total absorbed dose coefficients

	Present work			Lassman and Nosske [20]		Chittenden et al. [10]		Yoshida et al. [8]
Tissue	Alpha	Beta and gamma	Total	Alpha	Beta and gamma	Alpha	Beta and gamma	Total
Adrenals	2.07	0.29	2.35	3.2	0.24			0.06
Brain	1.95	0.21	2.15	3.2	0.18			0.05
Breast	1.95	0.12	2.07	3.2	0.16			0.02
Colon	2.11	2.71	4.82	9.5	25	0	47	21.9
Endost-BS	215.87	5.25	221.11	750	11	5378	21	761
Kidneys	24.90	1.16	26.07	3.4	0.24	6	< 1	2.00
Liver	34.39	1.50	35.88	36	1.5	2	< 1	1.87
Lungs	1.96	0.19	2.15	3.2	0.19			0.03
Muscle	1.93	0.18	2.12	3.2	0.2			0.06
Pancreas	2.07	0.30	2.36	3.2	0.22			0.06
Red marrow	30.75	3.08	33.82	72	5.5	408	9	91.6
Skin	2.07	0.11	2.18	3.2	0.16			0.03
SI-wall	2.09	0.26	2.35	3.2	0.39	0	5	5.42
Spleen	2.28	0.20	2.49	3.2	0.19			0.04
Stomach wall	2.09	0.26	2.35	3.2	0.21			0.08
Testes	2.10	0.12	2.21	3.2	0.18			0.03
Thymus	1.95	0.19	2.13	3.2	0.17			0.02
Thyroid	2.03	0.14	2.17	3.2	0.17			0.03
UB-wall	1.94	0.23	2.17	3.3	0.41	3	< 1	1.54

*Endost-BS*, endosteal cells; *SI-wall*, small intestine wall; *UB-wall*, urinary bladder (wall)

The relative contribution of each progeny to the absorbed dose of the organs which exhibit the highest values are given in Table 6: more than 99% of the absorbed dose is due to alpha and beta particles emitted from ^223^Ra and its progeny. Similar contributions of each ^223^Ra progeny to the bone lesions was reported by Murray et al. [27].

**Table 6:** Relative contribution of ^223^Ra and its progeny to the total absorbed dose of endosteum, red marrow, liver and kidneys (in %)

	^223^Ra	^219^Rn	^215^Po	^211^Pb	^211^Bi	^211^Po	^207^TI
Tissue							
Red marrow	19	24	26	4	22	0	4
Endost-BS	21	25	27	1	24	0	1
Liver	21	24	27	2	24	0	2
Kidneys	20	24	26	2	26	0	2

*Endost-BS*, endosteum (endosteal cells)

Of particular relevance to alpha particle therapy is a relative biological effectiveness (RBE) value which is suggested by some authors to account for the biological effect of high linear energy transfer (LET) radiation; Feinendegen and McClure [28] and the MIRD Committee [29] recommended for alpha particles a value between 3 and 5. Absorbed doses quoted in this article are for an RBE of unity.

## Discussion

### Biodistribution

In the last years, clinical trials using the radiopharmaceutical Xofigo® were undertaken to derive the distribution of ^223^Ra in the human body [8–11]; the pharmacokinetics were obtained by gamma spectrometry of the whole body, distinctive organs, blood, urine and faeces. An overview of the results of these studies is presented in Table 4 together with the results of the compartmental modelling study developed for this work. Parts of the results of the present work are in good agreement with the results of the clinical trials: this may reflect very fast plasma clearance, main uptake into bone (although by different proportion), small urinary excretion and large activity in the colon with main excretion of ^223^Ra in the faeces. Note that the urinary and faeces excretion within the Ra model has been considered on continuous excretion, which is not the case in reality. Similarly, the excretion data of the patients are given as cumulative values. The patients’ excretion rates are variable because of inter-individual metabolic differences and high variability in intestinal transit times [8]. In studies of Chittenden et al. [10], for example, activities of ^223^Ra in the small intestine are observed 4 h after intravenous administration, whereas the biokinetic model of ^223^Ra predicted by the ICRP does not include a specified compartment for *Small Intestine.* This is because due to physiological reasons, ^223^Ra is expected to be transferred into the small intestine from systemic circulation but for simplification; this pathway has been neglected because the dose to the small intestine was considered not to be relevant for radiation protection purposes. The increased initial uptake of ^223^Ra into the bones as reported in clinical studies [8, 10], is stated to be about 50-60%, whereas the modelled uptake of ^223^Ra into bones reached 30% after 4 h of injection. This reflects the difference between the ^223^Ra uptake into tumour lesions and normal bones. Taprogge and co-workers [30], who analysed data of six patients from Chittenden et al. [10], are in the process of developing a simple compartmental model for plasma, bone surfaces, small, and large intestines and excretion path that better fits to the patient data. Carrasquillo et al. [11] observed a small amount (not quantified) of activity in kidneys and liver, but only early after injection (< 4 h post-administration). In the present simulation, a low distribution of ^223^Ra in the liver and kidneys was derived at early times after administration (Fig. 4). For all other organs and tissues, both the clinical data and the modelled data showed no significant distribution of ^223^Ra and its progeny.

### Organ absorbed doses

The importance of patient dosimetry in radionuclide therapy and its limitations have been recognised by many authors and reviewed by Lassmann and Eberlein [31]. The present work is reporting recent developments combining new biokinetic and dosimetric modelling methods.

The results of the present work are summarised in Tables 5 and 6 showing the calculated organ absorbed doses for a reference adult male (73 kg, 176 cm height) after administration of ^223^Ra. The highest absorbed doses were found for the bone endosteum, liver, red marrow and kidneys. Mainly alpha particles emitted from ^223^Ra and its progeny deposit the absorbed dose in these organs; the isotopes ^219^Rn, ^215^Po and ^211^Bi contribute most to the dose. Similar contribution of the ^223^Ra progeny to bone lesion-absorbed doses was found in the study of Murray et al. [27].

The calculated dose coefficients for bone endosteum, red marrow and colon were lower than those reported from clinical data by Yoshida et al. [8] and Chittenden et al. [10]. On the contrary, the calculated kidney and liver doses of the present work were higher compared to those revealed by the clinical studies. The authors of these studies evaluated clinical imaging data and derived the cumulated activities through regions of interest (ROIs). For the dosimetry, the dosimetric tool OLINDA/EXM [32] was used, a software based on earlier dosimetric methods and SAFs derived on mathematical phantoms. Moreover, for the present work, independent biokinetics of each progeny were implemented, as shown in the supplement material. The resulted contributions to the doses of the red marrow, endosteum, kidneys and liver of the progeny such as ^219^Rn, ^215^Po and ^211^Bi, are in a similar range, i.e. about 20-27% (Table 6). In clinical studies, it is assumed that short-lived progeny deposits directly at the location of its parent radionuclide, whereas in the present work the biokinetics are independently modelled for each progeny. This may explain some of the disagreements observed.

It should be noted that data stemming from clinical studies cannot be compared directly with compartmental modelling calculations, as a specific tumour dosimetry, is not included in these models [33]; the latter can be, strictly speaking, considered valid for predicting the doses for a healthy, reference (i.e. average size) adult or for a dose assessment of a population and not for an individual patient. They are though very useful for predicting the trends of the radionuclide distribution and ensure adequate dose to the bone to fulfil the purpose of radiotherapeutic effect on bone metastasis and avoid radiotoxicity in other tissues. The results of the present study were compared to the results of the compartmental modelling of Lassmann and Nosske [20] and were found to be lower (skeletal doses by a factor of 2.3-3.4, colon dose by a factor of 7.2), except for the dose to kidneys, which was found to be higher by a factor of ca. 7. The dose coefficient to the liver shows a good agreement with the dose estimated by Lassmann and Nosske [2]. The discrepancies of dose coefficients may be due to the different pharmacokinetic models of radium applied. In this work, the new ICRP biokinetic model for ^223^Ra [12] was used whereas Lassmann and Nosske [20] used the previous ICRP model [13]. The latter model consists of only one liver compartment and has no specific kidney compartment. Further, the gastrointestinal tract (see ICRP Publication 30, [26]) includes the compartments *Upper Large Intestine* (*ULI*) and* Lower Large Intestine* (*LLI*) and there was no subdivision into the compartments *Right Colon* (*RC*), *Left Colon* (*LC*) and *Recto Sigmoid* (*RS*), as per the HATM [21]. In addition, several transfer rates of the previous ICRP radium model and its progeny are different compared to the new ICRP ^223^Ra model and its progeny. For example, in the previous radium model, the transfer coefficient from the compartments *Blood* to *Liver 1* was 0.35 d^−1^, whereas in the new radium model this value was increased to 4.2 d^−1^.

Moreover, the dosimetric framework of ICRP Publication 133 [19] used for the present work, presents several improvements in comparison to the former system [26] employed by the dosimetric software tools used by Lassmann and Nosske [20] and some clinical studies. For the skeletal dosimetry, which is of primary importance for this work, the target tissue is now the endosteum, defined as a 50-μm-thick layer covering the surfaces of the bone trabeculae in regions of trabecular spongiosa and those of the cortical surfaces of the medullary cavities within the shafts of all long bones [19, 23]. The endosteum as target tissue replaces the bone surfaces which were defined [34] as a single cell layer 10 μm in thickness, covering the surfaces of both the bone trabeculae and the Haversian canals of cortical bone. Moreover, the present calculation of the skeletal doses employs improved computational algorithms to estimate the absorbed dose to endosteum and red marrow [19]. Furthermore, differences in colon dose relate to changes in the dosimetric models, considering now the target cells in the colon walls to be located in a certain depth of the walls [21].

According to the findings of the present study (Table 5), after a simulated series of six treatments of 55 kBq/kg of ^223^Ra injected per treatment, which is often the recommended dosage to a patient, and for a patient with body mass of 73 kg [35], the absorbed dose to the endosteum would amount to 5.21 Gy and to the red marrow dose to 0.78 Gy, respectively. These values are consistent with the low haematological toxicity incidence reported [5]. Findings of clinical studies reviewed by Flux [36], showed much higher values of absorbed doses in bone surface and in red marrow, in the range from 54 to 303 Gy, and from 4 to 23 Gy, respectively. Similarly, Lassmann and Nosske [20] estimated for a male adult of 70 kg body mass [26] absorbed doses of 16 Gy, and 1.7 Gy in bone surface and red marrow, respectively, for a simulated series of six treatments, each of 50 kBq/kg ^223^Ra.

There are two further clinical studies for which quantitative imaging for dose estimation in metastatic bone lesions was applied: Pacilio et al. [37] estimated absorbed doses to bone lesions of 0.2-1.9 Gy after a single injection of 50 kBq/kg ^223^Ra. A study of Murray et al. [27] showed a wide range of absorbed doses across multiple sites of lesions (0.6-44.1 Gy) after a single administration of 110 kBq/kg ^223^Ra. According to the present work, absorbed dose of 0.87 Gy in the endosteum is estimated for a single simulated injection of 55 kBq/kg ^223^Ra, which is in agreement to the dose range reported by Pacilio et al. [37].

The comparison of the absorbed dose values estimated from modelling with values stemming from quantitative imaging in the clinical practice is challenging. Modelling is based on assumptions and algorithms developed for the reference size, healthy male but not the individual patient. On the other hand, clinical studies could involve large uncertainties in dose calculations based on imaging. Possibly, the limited count rates in imaging of ^223^Ra in liver and kidneys might explain the low dose coefficients estimated from quantification imaging compared to the much higher dose calculations in liver and kidneys of the present compartmental modelling. Flux [36] discussed the challenges in dosimetry: the heterogeneous uptake of the ^223^Ra (Xofigo®) in tissues and organs of patients, the difficulties to correctly determine the ROIs from the images and the quantification of ^223^Ra activities in the organs or tissues, the difficulties to estimate the time-integrated activity curves, or to assess lesion volumes. Additionally, a high variability of patients and different clinical techniques for imaging and dose calculations in the different clinics may lead to propagation of uncertainties in dose assessments.

Biokinetic modelling exhibits several uncertainties as well, such as uncertainty of biokinetic model structures, model parameters, experimental measurements for acquisition of biokinetic parameters such as transfer rates, uncertainty due to extrapolation of animal data to humans or due to the physiological variability of the individuals. These uncertainties could be propagated into the transfer rates of the biokinetic models and consequently to the modelled results, such as the time activity curves, time integrated activity coefficients and the calculated absorbed doses. Furthermore, improper implementation of the complicated biokinetic models in the modelling software could introduce a further uncertainty in absorbed doses.

The uncertainties and limitations of the dosimetric methodology are due to the anatomical and physics parameters employed for the estimation of the absorbed dose for internal emitters: limitations are present in the computational phantom representing the human anatomy and in the numerical procedures used to calculate the energy absorbed in the target tissues; the latter are associated with the transport of radiations in the body and the nuclear transformation processes that determine the energy and intensity of the emitted radiation. An extensive discussion of uncertainties in biokinetic and dosimetric models can be found at [38].

Accurate assessment of absorbed dose to the targeted regions is a requirement for the therapeutic purpose and for avoiding radiotoxicity to healthy surrounding tissues of a patient [39]. Population pharmacokinetic modelling is a useful tool to simulate the biokinetics of alpha-emitter radiopharmaceuticals, an alternative method, as imaging could be challenging in the clinical practice when alpha particles are involved. The quantification of excretion via population modelling could be used to optimise the therapy procedures by administering additional agents that could block the excretion pathways, allowing more time for circulation of the targeted ^223^Ra in the blood and increasing the possibility of bone uptake. The model could be improved by including a bone-site metastasis compartment. Moreover, the independent modelling of the progeny in the decay chain is a useful tool to understand the redistribution of progeny when alpha recoil effect occurs.

The population-valid biokinetic model and dose assessment method is a significant step towards patient-specific pharmacokinetic modelling and dosimetry for targeted radionuclide therapy. Specific biokinetics of clinical patients could be acquired as well as patient-specific voiding information for the bladder and colon which could then be implemented to be used for patient-specific dose evaluation.

## Conclusion

A new ICRP biokinetic model for radium and its progeny was applied for modelling the behaviour of the ^223^Ra, Xofigo® as an injected radiopharmaceutical to patients. Absorbed organ dose coefficients were estimated by applying the most recent ICRP dosimetric methods. The revealed similar trends concerning the plasma clearance and excretion via urine and faeces and highest uptake in bone of the modelled ^223^Ra data compared to clinical data showed the feasibility of applying the newly developed models in clinical trials. Discrepancies found in dose coefficients to the values reported from clinical studies and an earlier compartmental modelling study are attributed to the different biokinetic and dosimetric methods. Although the model was developed for a reference, healthy individual and applies, therefore, for a patient of average weight and height or a population, and not an individual patient, it is a very useful tool for optimisation and comparison of different modalities. Moreover, the model could be further developed for patient-specific dosimetry, implementing specific biokinetic, anatomical and physiological information of a patient.

## Supplementary Information

**Additional file 1: **Supplement: Schematic representation of the biokinetic models and transfer rates of the ^223^Ra decay chain used in this work. **Figure 1**. Systemic model of radon (^219^Rn) as progeny of ^223^Ra, used in the present work for biokinetic and dosimetric modelling [1]. Exch, exchangeable; nonexch, non-exchangeable; ST0, ST1, ST2 represent soft tissue (ST) with fast, intermediate, and slow turnover, respectively. **Table 1**. Model parameters of ^219^Rn as radioactive progeny of ^223^Ra: transfer coefficients k (per day) are taken from [1, 2]. **Figure 2**. Systemic model of polonium (^215^Po and ^211^Po respectively) as progeny of ^223^Ra, used in the present work for biokinetic and dosimetric modelling [1]. Exch, exchangeable; nonexch, non-exchangeable; ST0, ST1, ST2 represent soft tissue (ST) with fast, intermediate, and slow turnover, respectively; RBC, red blood cells. **Table 2**. Model parameters of ^215^Po, and ^211^Po respectively, as radioactive progeny of ^223^Ra: transfer coefficients k (per day) are taken from [1, 2]. The transfer rate from compartment *Plasma 1* to *ST*1 is lower than the value from [1] due to the additional compartment *Cortical Marrow* introduced for this work. **Figure 3**. Systemic model of lead (^211^Pb) as progeny of ^223^Ra used in the present work for biokinetic and dosimetric modelling [1]. Exch, exchangeable; nonexch, non-exchangeable; ST0, ST1, ST2 represent soft tissue (ST) with fast, intermediate, and slow turnover, respectively; RBC, red blood cells. **Table 3**. Model parameters of ^211^Pb as radioactive progeny of ^223^Ra: transfer coefficients *k* (per day) are taken from [1, 2]. Transfer rates from *Plasma* to all *Other Soft Tissue* compartments have lower values as those given at [1] due to the added compartments *Trabecular Marrow*, *Cortical Marrow*, *Spleen*, *Skin*, and *Testes* introduced for this work. **Figure 4**. Systemic model of bismuth (^211^Bi) as progeny of ^223^Ra used in the present work for biokinetic and dosimetric modelling [1]. Exch, exchangeable; nonexch, non-exchangeable; ST0, ST1, ST2 represent soft tissue (ST) with fast, intermediate, and slow turnover, respectively. **Table 4**. Model parameters of ^211^Bi as radioactive progeny of ^223^Ra: transfer coefficients k (per day) are taken from [1, 2]. The transfer rate from the compartment *Plasma* to *ST1* is lower than the value given at [1] due to the added compartments *Trabecular Marrow*, *Cortical Marrow*, *Spleen*, *Skin*, and *Testes*. **Figure 5**. Systemic model of thallium (^207^TI) as progeny of ^223^Ra used in the present work for biokinetic and dosimetric modelling [1]. Exch, exchangeable; nonexch, non-exchangeable; ST0, ST1, ST2 represent soft tissue (ST) with fast, intermediate, and slow turnover, respectively; RBC, red blood cells. **Table 5**. Model parameters for ^207^TI as radioactive progeny of ^223^Ra: transfer coefficients k (per day) are taken from [1, 2] and from human alimentary tract model (HATM) [2]

## Data Availability

The data used and evaluated during the current study are available from the corresponding author on reasonable request.

## Footnotes

1 The Epsilon Group, VA 22901, USA
