# Correction: Radiopharmacokinetic modelling and radiation dose assessment of ^223^Ra used for treatment of metastatic castration-resistant prostate cancer

**DOI:** 10.1186/s40658-024-00708-1

**Published:** 2025-01-23

**Authors:** Vera Höllriegl, Nina Petoussi-Henss, Kerstin Hürkamp, Juan Camilo Ocampo Ramos, Wei Bo Li

**Affiliations:** 1 Institute of Radiation Medicine, Helmholtz Zentrum München, German Research Center for Environmental Health, Ingolstädter Landstraße 1, 85764 Neuherberg, Germany; 2 Department of Medical Physics, Memorial Sloan Kettering Cancer Center, 1250 First Avenue, New York, NY 10065 USA

**Correction: EJNMMI Phys 8, 44 (2021)**

10.1186/s40658-021-00388-1

Following publication of the original article [1], the authors reported an error in Table 5.

In Table 5 of our paper [1], we incorrectly cited part of the work of Lassmann and Nosske [2] by giving the value of 3.6 mGy/MBq for the liver absorbed dose coefficient due to alphas after intravenous administration of ^223^Ra-chloride. The correct value should be 36 mGy/MBq. Consequently, the following text in page 13 of our paper [1] has to be amended:

“The results of the present study were compared to the results of the compartmental modelling of Lassmann and Nosske [20] and were found to be lower (skeletal doses by a factor of 2.3–3.4, colon dose by a factor of 7.2), except for the doses to liver and kidneys, which were found to be higher by a factor of ca. 7.”

Amended text:

“The results of the present study were compared to the results of the compartmental modelling of Lassmann and Nosske [20] and were found to be lower (skeletal doses by a factor of 2.3–3.4, colon dose by a factor of 7.2), except for the dose to kidneys, which was found to be higher by a factor of ca. 7. The dose coefficient to the liver shows a good agreement with the dose estimated by Lassmann and Nosske [2]”.

The authors thank Lassmann and Eberlein for pointing out this error [3] and apologize for the incorrect citation.

The original article [1] has been updated.
