# Supplementary material for: Radiopharmacokinetic modelling and radiation dose assessment of 223Ra used for treatment of metastatic castration-resistant prostate cancer
Source: EJNMMI Phys. 2021 Jun 2;8:44. doi: 10.1186/s40658-021-00388-1 (PMC8172819; doi:10.1186/s40658-021-00388-1)
Supplement: Supplementary file 1 — Additional file 1: Supplement: Schematic representation of the biokinetic models and transfer rates of the 223Ra decay chain used in this work. Figure 1. Systemic model of radon (219Rn) as progeny of 223Ra, used in the present work for biokinetic and dosimetric modelling [1]. Exch, exchangeable; nonexch, non-exchangeable; ST0, ST1, ST2 represent soft tissue (ST) with fast, intermediate, and slow turnover, respectively. Table 1. Model parameters of 219Rn as radioactive progeny of 223Ra: transfer coefficients k (per day) are taken from [1, 2]. Figure 2. Systemic model of polonium (215Po and 211Po respectively) as progeny of 223Ra, used in the present work for biokinetic and dosimetric modelling [1]. Exch, exchangeable; nonexch, non-exchangeable; ST0, ST1, ST2 represent soft tissue (ST) with fast, intermediate, and slow turnover, respectively; RBC, red blood cells. Table 2. Model parameters of 215Po, and 211Po respectively, as radioactive progeny of 223Ra: transfer coefficients k (per day) are taken from [1, 2]. The transfer rate from compartment Plasma 1 to ST1 is lower than the value from [1] due to the additional compartment Cortical Marrow introduced for this work. Figure 3. Systemic model of lead (211Pb) as progeny of 223Ra used in the present work for biokinetic and dosimetric modelling [1]. Exch, exchangeable; nonexch, non-exchangeable; ST0, ST1, ST2 represent soft tissue (ST) with fast, intermediate, and slow turnover, respectively; RBC, red blood cells. Table 3. Model parameters of 211Pb as radioactive progeny of 223Ra: transfer coefficients k (per day) are taken from [1, 2]. Transfer rates from Plasma to all Other Soft Tissue compartments have lower values as those given at [1] due to the added compartments Trabecular Marrow, Cortical Marrow, Spleen, Skin, and Testes introduced for this work. Figure 4. Systemic model of bismuth (211Bi) as progeny of 223Ra used in the present work for biokinetic and dosimetric modelling [1]. Exch, exchangeable; nonexch, non-ex [file 40658_2021_388_MOESM1_ESM.docx]

**Supplement**:

**Schematic representation of the biokinetic models and transfer rates of the ^223^Ra decay chain used in this work**


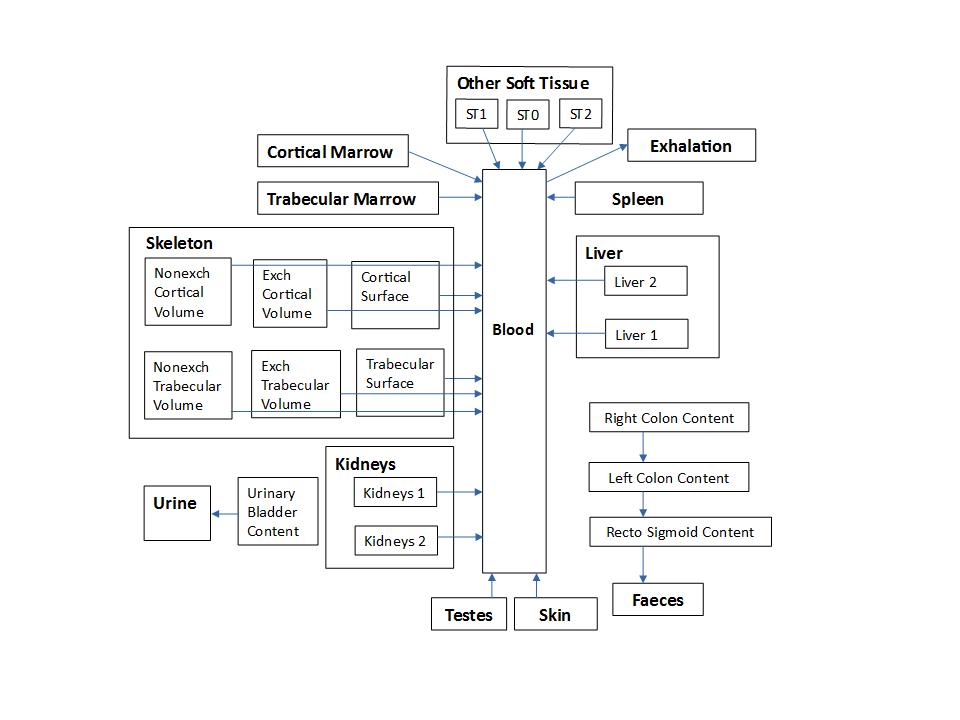


**Fig. 1** Systemic model of radon (^219^Rn) as progeny of ^223^Ra, used in the present work for biokinetic and dosimetric modelling [1]. Exch, exchangeable; nonexch, non-exchangeable; ST0, ST1, ST2 represent soft tissue (ST) with fast, intermediate, and slow turnover, respectively

**Table 1** Model parameters of ^219^Rn as radioactive progeny of ^223^Ra: transfer coefficients k (per day) are taken from [1, 2].

| from | to | k (d^-1^) |
| --- | --- | --- |
| Blood | Exhalation | 1000 |
| ST0 | Blood | 33.27 |
| ST1 | Blood | 33.27 |
| ST2 | Blood | 33.27 |
| Cortical bone surface | Blood | 100 |
| Exch cortical bone volume | Blood | 1.5 |
| Nonexch cortical bone volume | Blood | 0.36 |
| Trabecular bone surface | Blood | 100 |
| Exch trabecular bone volume | Blood | 1.5 |
| Nonexch trabecular bone volume | Blood | 0.36 |
| Trabecular marrow | Blood | 33.27 |
| Cortical marrow | Blood | 33.27 |
| Liver 1 | Blood | 33.27 |
| Liver 2 | Blood | 33.27 |
| Kidneys 1 | Blood | 33.27 |
| Kidneys 2 | Blood | 33.27 |
| Skin | Blood | 33.27 |
| Spleen | Blood | 33.27 |
| Testes | Blood | 33.27 |
| Right colon content | Left colon content | 2 |
| Left colon content | Recto sigmoid content | 2 |
| Recto sigmoid content | Faeces | 2 |
| Urinary bladder content | Urine | 12 |

Exch, exchangeable; nonexch, non-exchangeable; ST0, ST1, ST2 represent soft tissue with fast, intermediate, and slow turnover, respectively


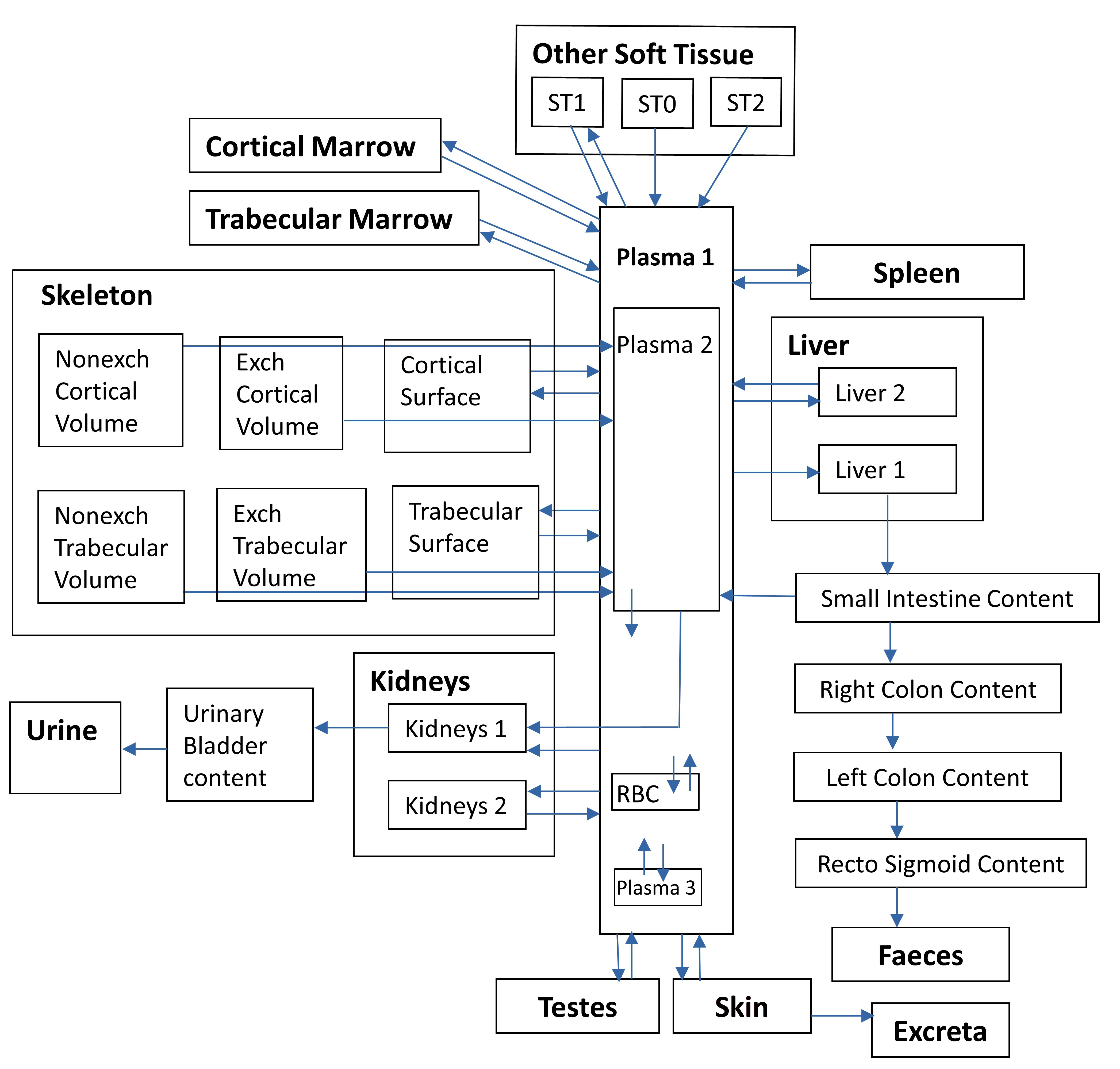


**Fig. 2** Systemic model of polonium (^215^Po and ^211^Po respectively) as progeny of ^223^Ra, used in the present work for biokinetic and dosimetric modelling [1]. Exch, exchangeable; nonexch, non-exchangeable; ST0, ST1, ST2 represent soft tissue (ST) with fast, intermediate, and slow turnover, respectively; RBC, red blood cells

**Table 2** Model parameters of ^215^Po, and ^211^Po respectively, as radioactive progeny of ^223^Ra: transfer coefficients k (per day) are taken from [1, 2]. The transfer rate from compartment *Plasma 1* to *ST1* is lower than the value from [1] due to the additional compartment *Cortical Marrow* introduced for this work

| from | to | k (d^-1^) |
| --- | --- | --- |
| Plasma 2 | Plasma 1 | 800 |
| Plasma 2 | Kidneys 1 | 200 |
| Plasma 1 | Plasma 3 | 4 |
| Plasma 1 | RBC | 6 |
| Plasma 1 | ST1 | 31.02 |
| ST0 | Plasma 1 | 0.099 |
| ST1 | Plasma 1 | 0.099 |
| ST2 | Plasma 1 | 0.099 |
| Plasma 3 | Plasma 1 | 0.099 |
| Plasma 1 | Cortical bone surface | 0.75 |
| Plasma 1 | Trabecular bone surface | 0.75 |
| Plasma 1 | Kidneys 1 | 5 |
| Plasma 1 | Kidneys 2 | 5 |
| Plasma 1 | Liver 1 | 17.5 |
| Plasma 1 | Liver 2 | 17.5 |
| RBC | Plasma 1 | 0.099 |
| Liver 1 | Small intestine content | 0.139 |
| Liver 2 | Plasma 1 | 0.099 |
| Cortical bone surface | Plasma 1 | 0.0231 |
| Nonexch cortical bone volume | Plasma 2 | 0.0000821 |
| Exch cortical bone volume | Plasma 2 | 0.0000821 |
| Trabecular bone surface | Plasma 1 | 0.0231 |
| Nonexch trabecular bone volume | Plasma 2 | 0.000493 |
| Exch trabecular bone volume | Plasma 2 | 0.000493 |
| Trabecular marrow | Plasma 1 | 0.099 |
| Plasma 1 | Trabecular marrow | 4 |
| Cortical marrow | Plasma 1 | 0.099 |
| Plasma 1 | Cortical marrow | 1.33 |
| Kidneys 1 | Urinary bladder content | 0.173 |
| Kidneys 2 | Plasma 1 | 0.099 |
| Plasma 1 | Skin | 5 |
| Plasma 1 | Spleen | 2 |
| Plasma 1 | Testes | 0.1 |
| Skin | Plasma 1 | 0.00693 |
| Skin | Excreta | 0.00693 |
| Spleen | Plasma 1 | 0.099 |
| Testes | Plasma 1 | 0.0139 |
| Small intestine (SI) content | Plasma 2 | 0.667^a^ |
| SI content | Right colon content | 6 |
| Right colon content | Left colon content | 2 |
| Left colon content | Recto sigmoid content | 2 |
| Recto sigmoid content | Faeces | 2 |
| Urinary bladder content | Urine | 12 |

Exch, exchangeable; nonexch, non-exchangeable; ST0, ST1, ST2 represent soft tissue with fast, intermediate, and slow turnover, respectively; RBC, red blood cells;

^a^based on the transfer of 6 d^-1^ from SI content to Right Colon content [2] and on a respective fractional absorption factor of 0.1 [1]


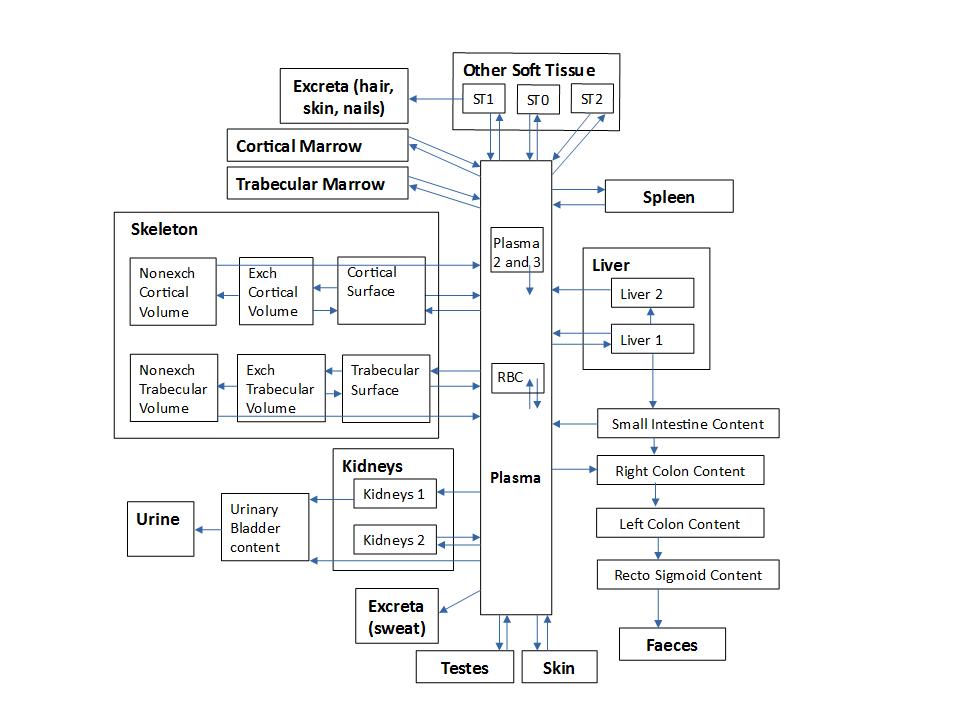


**Fig. 3** Systemic model of lead (^211^Pb) as progeny of ^223^Ra used in the present work for biokinetic and dosimetric modelling [1]. Exch, exchangeable; nonexch, non-exchangeable; ST0, ST1, ST2 represent soft tissue (ST) with fast, intermediate, and slow turnover, respectively; RBC, red blood cells

**Table 3** Model parameters of ^211^Pb as radioactive progeny of ^223^Ra: transfer coefficients *k* (per day) are taken from [1, 2]. Transfer rates from *Plasma* to all *Other* *Soft Tissue* compartments have lower values as those given at [1] due to the added compartments *Trabecular* *Marrow*, *Cortical Marrow*, *Spleen*, *Skin*, and *Testes* introduced for this work

| from | To | *k* (d^-1^) |
| --- | --- | --- |
| Plasma | Excreta (sweat) | 0.42 |
| Plasma | ST0 | 21.606 |
| Plasma | ST1 | 0.6325 |
| Plasma | ST2 | 0.1365 |
| Plasma | Cortical bone surface | 3.89 |
| Plasma | Trabecular bone surface | 4.86 |
| Plasma | Kidneys 1 | 2.45 |
| Plasma | Kidneys 2 | 0.0245 |
| Plasma | Liver 1 | 4.9 |
| Plasma | Right colon content | 0.7 |
| Plasma | Urinary bladder content | 1.75 |
| RBC | Plasma | 0.139 |
| Plasma | RBC | 28.00 |
| Liver 1 | Plasma | 0.0312 |
| Liver 2 | Plasma | 0.0019 |
| Liver 1 | Small intestine content | 0.0312 |
| Liver 1 | Liver 2 | 0.00693 |
| ST0 | Plasma | 7.39 |
| ST1 | Plasma | 0.00416 |
| ST1 | Excreta (skin, hair, nails) | 0.00277 |
| ST2 | Plasma | 0.00038 |
| Cortical bone surface | Plasma | 0.5 |
| Cortical bone surface | Exch cortical bone volume | 0.5 |
| Exch cortical bone volume | Cortical bone surface | 0.0185 |
| Exch cortical bone volume | Nonexch cortical bone volume | 0.0046 |
| Nonexch cortical bone volume | Plasma | 0.0000821 |
| Trabecular bone surface | Plasma | 0.5 |
| Trabecular bone surface | Exch trabecular bone volume | 0.5 |
| Exch trabecular bone volume | Trabecular bone surface | 0.0185 |
| Exch trabecular bone volume | Nonexch trabecular bone volume | 0.0046 |
| Nonexch trabecular bone volume | Plasma | 0.000493 |
| Trabecular marrow | Plasma | 0.00189 |
| Plasma | Trabecular marrow | 0.42 |
| Cortical marrow | Plasma | 0.00189 |
| Plasma | Cortical marrow | 0.14 |
| Kidneys 1 | Urinary bladder content | 0.139 |
| Kidneys 2 | Plasma | 0.0019 |
| Plasma | Skin | 0.14 |
| Plasma | Spleen | 0.021 |
| Plasma | Testes | 0.0035 |
| Skin | Plasma | 0.00189 |
| Spleen | Plasma | 0.00189 |
| Testes | Plasma | 0.00189 |
| Small intestine (SI) content | Plasma | 1.5^a^ |
| SI content | Right colon content | 6 |
| Right colon content | Left colon content | 2 |
| Left colon content | Recto sigmoid content | 2 |
| Recto sigmoid content | Faeces | 2 |
| Urinary bladder content | Urine | 12 |
| Plasma 2*, Plasma 3* | Plasma | 1000 |

Exch, exchangeable; nonexch, non-exchangeable; ST0, ST1, ST2 represent soft tissue with fast, intermediate, and slow turnover, respectively; RBC, red blood cells

^a^based on the transfer of 6 d^-1^ from SI content to Right Colon content [2] and on the fractional absorption factor of 0.2 [3]

*according to [1], lead produced by radioactive decay in a blood compartment which is not identifiable with a blood compartment of the lead model, is assumed to transfer to plasma at the rate of 1000 d^-1^


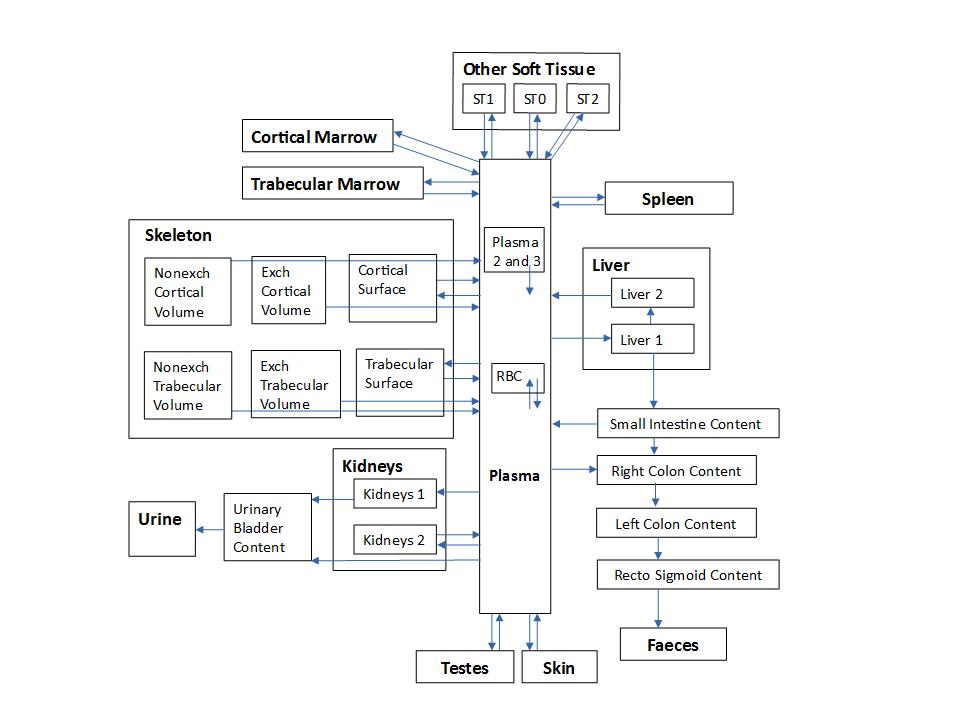


**Fig. 4** Systemic model of bismuth (^211^Bi) as progeny of ^223^Ra used in the present work for biokinetic and dosimetric modelling [1]. Exch, exchangeable; nonexch, non-exchangeable; ST0, ST1, ST2 represent soft tissue (ST) with fast, intermediate, and slow turnover, respectively

**Table 4** Model parameters of ^211^Bi as radioactive progeny of ^223^Ra: transfer coefficients k (per day) are taken from [1, 2]. The transfer rate from the compartment *Plasma* to *ST1* is lower than the value given at [1] due to the added compartments *Trabecular* *Marrow*, *Cortical Marrow*, *Spleen*, *Skin*, and *Testes*

| from | to | k (d^-1^) |
| --- | --- | --- |
| Plasma | ST0 | 300 |
| Plasma | ST1 | 3.477 |
| Plasma | ST2 | 1.3 |
| Plasma | Cortical bone surface | 2.5 |
| Plasma | Trabecular bone surface | 2.5 |
| Plasma | Kidneys 1 | 30 |
| Plasma | Kidneys 2 | 5 |
| Plasma | Liver 1 | 30 |
| Plasma | Right colon content | 4 |
| Plasma | Urinary bladder content | 20 |
| RBC | Plasma | 0.173 |
| Plasma | RBC | 0.5 |
| Liver 2 | Plasma | 0.0693 |
| Liver 1 | Small intestine content | 0.208 |
| Liver 1 | Liver 2 | 0.139 |
| ST0 | Plasma | 66.54 |
| ST1 | Plasma | 0.0347 |
| ST2 | Plasma | 0.00116 |
| Cortical bone surface | Plasma | 0.0347 |
| Trabecular bone surface | Plasma | 0.0347 |
| Trabecular marrow | Plasma | 0.0347 |
| Plasma | Trabecular marrow | 0.3 |
| Cortical marrow | Plasma | 0.0347 |
| Plasma | Cortical marrow | 0.1 |
| Kidneys 1 | Urinary bladder content | 0.693 |
| Kidneys 2 | Plasma | 0.139 |
| Plasma | Skin | 0.3 |
| Plasma | Spleen | 0.02 |
| Plasma | Testes | 0.003 |
| Skin | Plasma | 0.0347 |
| Spleen | Plasma | 0.0347 |
| Testes | Plasma | 0.0347 |
| Nonexch cortical bone volume | Plasma | 0.0000821 |
| Exch cortical bone volume | Plasma | 0.0000821 |
| Nonexch trabecular bone volume | Plasma | 0.000493 |
| Exch trabecular bone volume | Plasma | 0.000493 |
| Small intestine (SI) content | Plasma | 0.3158^a^ |
| SI content | Right colon content | 6 |
| Right colon content | Left colon content | 2 |
| Left colon content | Recto sigmoid content | 2 |
| Recto sigmoid content | Faeces | 2 |
| Urinary bladder content | Urine | 12 |
| Plasma 2*, Plasma 3* | Plasma | 1000 |

Exch, exchangeable; nonexch, non-exchangeable; ST0, ST1, ST2 represent soft tissue with fast, intermediate, and slow turnover, respectively; RBC, red blood cells

^a^based on the transfer of 6 d^-1^ from SI content to Right Colon content [2] and on the fractional absorption factor of 0.05 [4]

*according to [1], bismuth produced by radioactive decay in a blood compartment which is not identifiable with a blood compartment of the bismuth model, is assumed to transfer to plasma with the rate of 1000 d^-1^

**
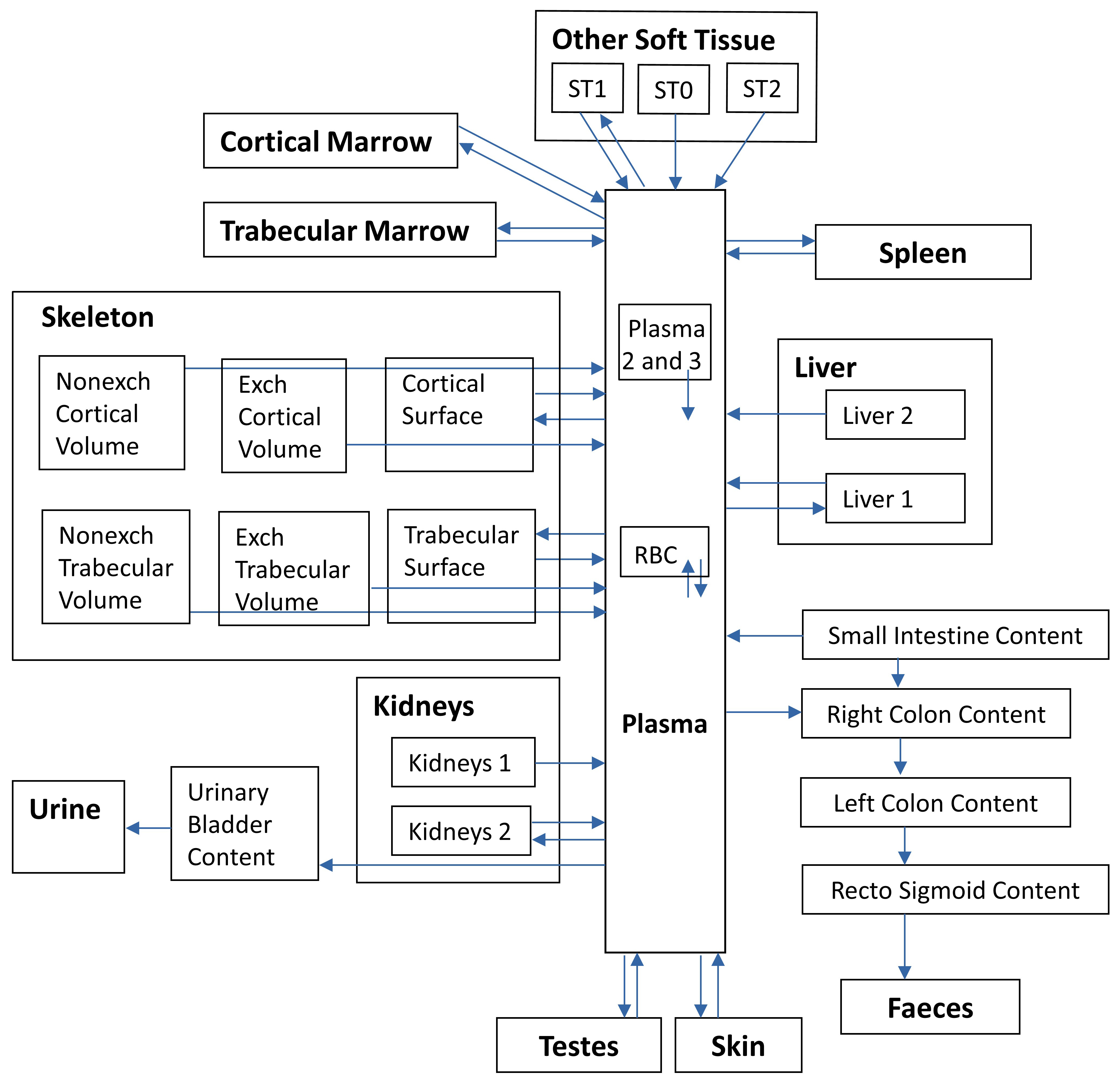
**

**Fig. 5** Systemic model of thallium (^207^TI) as progeny of ^223^Ra used in the present work for biokinetic and dosimetric modelling [1]. Exch, exchangeable; nonexch, non-exchangeable; ST0, ST1, ST2 represent soft tissue (ST) with fast, intermediate, and slow turnover, respectively; RBC, red blood cells

**Table 5** Model parameters for ^207^TI as radioactive progeny of ^223^Ra: transfer coefficients k (per day) are taken from [1, 2] and from human alimentary tract model (HATM) [2]

| from | to | k (d^-1^) |
| --- | --- | --- |
| Plasma | ST1 | 130.8 |
| ST0 | Plasma | 2.5 |
| ST1 | Plasma | 2.5 |
| ST2 | Plasma | 2.5 |
| Plasma | Cortical bone surface | 15 |
| Plasma | Trabecular bone surface | 15 |
| Plasma | Kidneys 2 | 10 |
| Plasma | Liver 1 | 10 |
| Plasma | Right colon content | 3.5 |
| Plasma | Urinary bladder content | 1.5 |
| Plasma | RBC | 5 |
| RBC | Plasma | 3.7 |
| Liver 1 | Plasma | 2.5 |
| Liver 2* | Plasma | 2.5 |
| Cortical bone surface | Plasma | 2.5 |
| Trabecular bone surface | Plasma | 2.5 |
| Nonexch cortical bone volume | Plasma | 0.0000821 |
| Exch cortical bone volume | Plasma | 0.0000821 |
| Nonexch trabecular bone volume | Plasma | 0.000493 |
| Exch trabecular bone volume | Plasma | 0.000493 |
| Trabecular marrow | Plasma | 2.5 |
| Plasma | Trabecular marrow | 3 |
| Cortical marrow | Plasma | 2.5 |
| Plasma | Cortical marrow | 1 |
| Kidneys 2 | Plasma | 2.5 |
| Kidneys 1* | Plasma | 2.5 |
| Plasma | Skin | 5 |
| Plasma | Spleen | 0.4 |
| Plasma | Testes | 0.6 |
| Skin | Plasma | 2.5 |
| Spleen | Plasma | 2.5 |
| Testes | Plasma | 2.5 |
| Small intestine (SI) content | Plasma | 594^a^ |
| SI content | Right colon content | 6 |
| Right colon content | Left colon content | 2 |
| Left colon content | Recto sigmoid content | 2 |
| Recto sigmoid content | Faeces | 2 |
| Urinary bladder content | Urine | 12 |
| Plasma 2**, Plasma 3** | Plasma | 1000 |

Exch, exchangeable; nonexch, non-exchangeable; ST0, ST1, ST2 represent soft tissue with fast, intermediate, and slow turnover, respectively; RBC, red blood cells

^a^based on the transfer of 6 d^-1^ from SI content to Right Colon content [2] and on the fractional absorption factor of 0.99 [5]

*according to [1], thallium produced by radioactive decay in a soft tissue compartment which is not identifiable with a compartment of the thallium model, is assumed to transfer to plasma at the rate of 2.5 d^-1^

**according to [1], thallium produced by radioactive decay in a blood compartment which is not identifiable with a blood compartment of the thallium model, is assumed to transfer to plasma at the rate of 1000 d^-1^

References:

1. ICRP. Occupational intakes of radionuclides: Part 3. ICRP Publication 137: International Commission of Radiological Protection; 2017.

2. ICRP. Human alimentary tract model for radiological protection. ICRP Publication 100. Oxford, UK: Elsevier; 2006.

3. ICRP. Age-dependent doses to members of the public from intake of radionuclides: Part 2: Ingestion dose coefficients. ICRP Publication 67. Oxford, UK: Pergamon Press; 1993.

4. ICRP. Limits for intakes of radionuclides by workers. Part 2. ICRP Publication 30. Oxford, UK: Pergamon Press; 1980.

5. Leggett RW, Ansoborlo E, Bailey M, Gregoratto D, Paquet F, Taylor DM. Biokinetic data and models for occupational intake of lanthanoids. Int J Radiat Biol. 2014; 90:996-1010.
